# Pantothenic acid – a scoping review for Nordic Nutrition Recommendations 2023

**DOI:** 10.29219/fnr.v67.10255

**Published:** 2023-12-13

**Authors:** Riitta Freese, Tonje E. Aarsland, Maja Bjørkevoll

**Affiliations:** 1 Department of Food and Nutrition, University of Helsinki, Helsinki, Finland; 2 Department of Global Public Health and Primary Care, University of Bergen, Bergen, Norway; 3 Centre for International Health, University of Bergen, Bergen, Norway

**Keywords:** pantothenic acid, Coenzyme A, requirements, nutrition recommendations, Nordic countries

## Abstract

Pantothenic acid, also referred to as vitamin B_5_, is a water-soluble vitamin that has essential functions in the body as a component of coenzyme A (CoA) and acyl carrier protein (ACP). It is widely distributed in animal and plant-source foods. Nutritional deficiency of pantothenic acid is rare and toxicity negligible. Information on pantothenic acid intakes in the Nordic countries is limited and biomarker data from Nordic and Baltic populations is missing. Due to a lack of data, no dietary reference values (DRVs) were given for pantothenic acid in the Nordic Nutrition Recommendations (NNR) since 2012. The aim of this scoping review was to examine recent evidence relevant for updating the DRVs for NNR2023. Scientific literature since 2012 on associations of pantothenic acid with health-related issues in Nordic and Baltic countries was searched. No health concerns related to pantothenic acid were identified.

## Popular scientific summary

- Pantothenic acid is a water-soluble vitamin (also known as vitamin B_5_), essential for various metabolic reactions as a component of coenzyme A (CoA) and acyl carrier protein.
- Pantothenic acid is widely distributed in both animal and plant foods, but data on pantothenic acid intake in Nordic and Baltic populations is lacking.
- Urinary pantothenic acid excretion is considered the most reliable indicator of vitamin status, but no cut-off value for adequacy is established.
- Deficiency is rare; burning feet syndrome is one symptom of deficiency.
- Evidence for effects on chronic diseases is sparse.

Pantothenic acid (dihydroxy-b,b-dimethylbutyryl-b-alanine) is a water-soluble vitamin that belongs to the group of B-vitamins. As part of coenzyme A (CoA) and acyl carrier protein (ACP), pantothenic acid is essential to a large number of metabolic reactions. About 4% of cellular enzymes use CoA or its derivatives as a substrate. ACP is needed in fatty acid synthesis. Pantothenic acid is synthesized by microbes and plants, and it is widely distributed in foods; the name derives from the Greek word ‘pantos’, meaning ‘from everywhere’. The main dietary forms are CoA and protein- or peptide-bound phosphopantetheine. Information on the bioavailability of dietary pantothenic acid is scarce. In the body, the largest pools of pantothenic acid are in the form of CoA in mitochondria. The main excretion route is in urine as pantothenic acid.

Pantothenic acid is not part of food composition tables in most Nordic countries and information on pantothenic acid intake is limited. However, nutritional deficiency of pantothenic acid is rare and toxicity negligible. The aim of this scoping review is to describe the evidence for the role of pantothenic acid for health-related outcomes and evaluate whether this evidence may be the basis for establishing dietary reference values (DRVs) for the NNR2023 (Box 1).

Box 1 The role of the present scoping review in the NNR2023 process

- This article is one of many scoping reviews commissioned as part of the Nordic Nutrition Recommendations 2023 (NNR2023) project (1)
- The articles are included in the extended NNR2023 report but, for transparency, these scoping reviews are also published in Food & Nutrition Research
- The scoping reviews have been peer reviewed by independent experts in the research field according to the standard procedures of the journal.
- The scoping reviews have also been subjected to public consultations (see report to be published by the NNR2023 project).
- The NNR2023 committee has served as the editorial board.
- While these articles are a main fundament, the NNR2023 committee has the sole responsibility for setting dietary reference values in the NNR2023 project.

## Methods

This scoping review follows the protocol developed within the NNR2023 (1). The sources of evidence used in the scoping review follow the eligibility criteria described earlier (2). No *de novo* NNR2023 systematic reviews relevant for this review were conducted (3).

The main literature search for this review was performed on November 22, 2021 in MEDLINE with the search string: (‘pantothenic acid’[MeSH Terms]) AND (‘2011’[PDAT] : ‘3000’[PDAT]) AND review[Publication Type]) AND Humans[Filter]. The number of hits was 21. Based on the title, nine articles were picked up, of which six were considered relevant based on the full articles (4–9). Another search was carried out with string (‘pantothenic acid’[title]) AND (‘2011’[PDAT]: ‘3000’[PDAT]) AND Humans[Filter]. The number of hits was 18. Based on the title and abstracts, five articles were picked up of which three were considered relevant based on the full articles (10–12). None of the relevant articles was a systematic review. One article (13) was identified using the ‘Cited by’ feature of PubMed.

To update the Physiology and Health outcomes sections, relevant up-to-date textbooks were consulted (14–16). The previously published pantothenic acid recommendations or opinions from NNR2012 (17), Institute of Medicine (from 2015 onwards National Academy of Sciences, Engineering, and Medicine, NASEM) (18) and European Food Safety Authority (EFSA) (19, 20) were also used in the present scoping review.

## Physiology

Pantothenic acid is the trivial name of the compound dihydroxy-b,b-dimethylbutyryl-b-alanine. The metabolically active forms of pantothenic acid are CoA and ACP. Pantothenic acid is synthesized in plants and micro-organisms but animals require a dietary source.

### Digestion, absorption, transport, excretion and bioavailability

The main forms of dietary pantothenic acid are CoA and protein- or peptide-bound 4’-phosphopantetheine. In the small intestinal lumen, 4’-phosphopantetheine is released from CoA and ACP by hydrolases, dephosphorylated to pantetheine and hydrolyzed to pantothenic acid by pantetheinase (14, 16). Pantothenic acid is taken up in the enterocytes by the same SLC5A6 (solute carrier family 5 member 6) /SMVT (sodium-dependent multivitamin transporter) carrier as biotin and lipoic acid. Pantothenic acid is transported in the circulation in the free form and in red blood cells. The uptake to erythrocytes occurs by passive diffusion. In most other tissues the uptake is mediated by SMVT (14, 15). The SLC5A6/SMVT carrier takes pantothenic acid also across the blood–brain barrier (11).

The largest pools of pantothenic acid in tissues are in the form of CoA in mitochondria. The synthesis of CoA is a multistep process where the first step (phosphorylation to 4’-phosphopantetheine) is the rate-limiting reaction. Mutation of the kinase enzyme needed in this step results in pantothenate kinase-associated neurodegeneration (PKAN), an inborn error of CoA biosynthesis that leads to iron accumulation in the brain (4). Pantothenic acid can be liberated from CoA and ACP and reutilized for coenzyme synthesis (‘recycling’) (6, 13, 14). The extracellular pantetheinase enzymes that liberate pantothenic acid are coded by vanin genes (vascular-non-inflammatory molecules) and their roles in inflammation, oxidative stress, cell migration and numerous diseases are actively studied (6–8, 13). Vanin 1 concentration in the circulation increases during fasting, which probably serves to recycle pantothenic acid for CoA synthesis within cells (13).

Pantothenic acid is excreted in the urine mainly as free unmetabolized pantothenic acid. At physiological concentrations, pantothenic acid is largely reabsorbed in kidney tubules (15).

The bioavailability of pantothenic acid from foods has not been widely investigated. A bioavailability range of 40–60% from the American diet has been indicated (14). Pantothenic acid may be synthesized by gut microbiota. Furthermore, colonocytes express the same SMVT carrier as enterocytes in the small intestine. The extent to which the synthesis in the colon may contribute to human pantothenic acid intake is, however, not known (14, 21).

### Metabolic functions

**Fig. 1:**
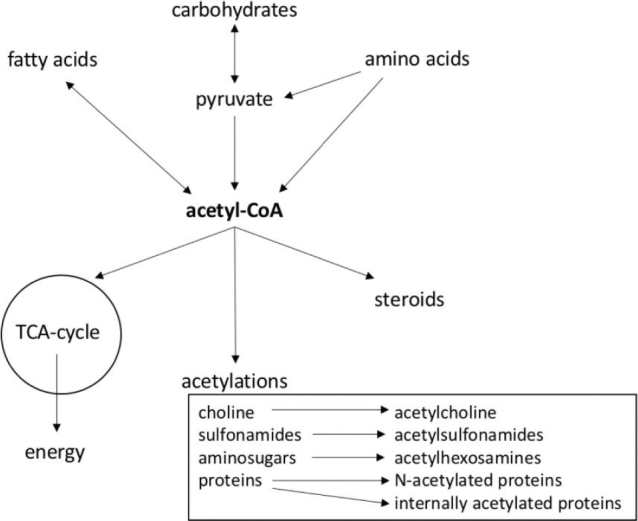
Acetyl-coenzyme A (Acetyl-CoA) has a central role in metabolism (modified from reference 14). TCA = tricarboxylic acid.

As a component of CoA, pantothenic acid is needed in a large number of metabolic reactions. About 4% of cellular enzymes use CoA or its thioester derivatives as a substrate. The central role of acetyl-CoA is illustrated in Fig. 1. ACP is needed in fatty acid synthesis. It is synthesized by binding 4’-phosphopantetheine to a serine residue of the ACP domain of the large fatty acid synthase complex (13).

## Assessment of nutrient status

Urinary pantothenic acid excretion reflects recent pantothenic acid intake and is considered the most reliable indicator of vitamin status (16, 19). Positive linear correlations between intakes and pantothenic acid concentrations in 24-h urine have been reported in small-scale interventions (reviewed in 19). Moderate correlations have been observed also between pantothenic acid intakes and its concentrations in whole blood or erythrocytes (19). However, population-level data on pantothenic acid biomarkers are lacking, and no cut-off values for pantothenic acid adequacy or insufficiency have been established (19). Concentrations <1 μmol/L in whole blood and urinary excretion <1 mg/d are considered low (16).

## Dietary intake in Nordic and Baltic countries

Pantothenic acid is widely distributed in foods of both animal and vegetable origin. Foods rich in pantothenic acid include organ meats, eggs, seafood, cheese, mushrooms, legumes, whole grains, vegetables, and nuts. Pantothenic acid may also be added to foods, supplements and formulas (22, 23). Few of the Nordic and Baltic countries have available information on the concentration of pantothenic acid in foods. Thus, there is little knowledge of the intake of pantothenic acid in these countries. From data collected from eight European countries, the mean/median intake of pantothenic acid has been estimated to be 3.2–6.3 mg/day in men and women aged 18–65 years (19). In children aged 3–12 years and 11–19 years, mean/median intakes of 3–5.7 mg/day and 3–7.2 mg/day have been reported, respectively (19).

## Health outcomes relevant for Nordic and Baltic countries

### Deficiency

Due to the widespread occurrence of pantothenic acid in foods, it is unlikely that deficiency is common in the general population. Deficiency is more likely to occur in conjunction with multiple nutrient deficiencies (14). In small experimental studies lasting 8–12 weeks deficiency has been observed in people given a diet free of pantothenic acid or administered a pantothenic acid antagonist (24–26). Symptoms of deficiency include burning feet syndrome, with experience of numbness and burning in the feet. Other symptoms of deficiency include fatigue, weakness, restlessness, sleep disturbances, muscle cramps, and irritability (14, 18).

### Upper intake levels and toxicity

The toxicity of pantothenic acid has not been observed from food sources. Clinical studies using doses up to 2 g of pantothenic acid per day has not proved a health risk for the general population (20). Daily intakes of 10–20 g have been related to water retention and occasional diarrhea. No tolerable upper intake level (UL) has been established by the Scientific Committee on Food (SCF) due to the lack of systematic oral dose response intake studies (20).

### Nutrient-related chronic diseases

Research on the relationship between pantothenic acid and chronic disease is limited. However, CoA metabolism and thus also pantothenic acid may have a role in the inflammatory processes (6, 7, 13). A prospective cohort study on men and women above 40 years of age from South Korea demonstrated an inverse relationship between C reactive protein (CRP) concentrations in serum and pantothenic acid intake (10). The average pantothenic acid intake in the study was 4.5 mg/day and 4.0 mg/day for men and women, respectively. Further studies are needed to investigate the relationship between pantothenic acid intake and inflammation-mediated chronic diseases.

The neurodegenarative disease PKAN, which is due to mutations in the pantothenate kinase enzyme 2, illustrates that pantothenic acid has an important role in brain health and integrity (5). Cerebral white matter contains large amounts of pantothenic acid (12). Pantothenic acid content in post-mortem brain samples from patients with Alzheimer´s disease (AD) has been shown to be lower than in healthy controls (12), which may be explained by dysregulation of pantothenic acid metabolism or uptake rather than dietary deficiency. Pantothenic acid supplementation may be of benefit in the treatment of PKAN. High doses (up to 2–5 g/d) for at least 3 months has been recommended for all patients with PKAN. If the patient does not perceive any benefit from the treatment, the supplementation should be discontinued (5).

### Other health-related aspects

Pharmacological derivatives of pantothenic acid are used topically in skin treatment due to their wound healing potential (4, 9).

## Requirement and recommended intakes

Due to a lack of data, neither requirements nor recommended intake of pantothenic acid were established in the NNR2012 (17). EFSA suggested adequate intakes (AIs) based on dietary intake data in several European countries with no sign of deficiency. The proposed AI is 5 mg/day for adults and children above 11 years of age (19). For infants and younger children, 3 and 4 mg is proposed, respectively, after appropriate extrapolation and scaling. Due to excretion through breastmilk, EFSA increased AIs during lactation (7 mg/day) (19). NASEM (former Institute of Medicine) (18) also applied AIs for the same reason as EFSA, with a slightly increased AI during pregnancy (6 mg/day).

### Data gaps for future research

The concentration of pantothenic acid in foods should be analyzed and incorporated into the Nordic food composition tables in order to estimate dietary intakes in the Nordic populations and requirements of this vitamin.
